# Active Tobacco Smoke Exposure in Utero and Concentrations of Hepcidin and Selected Iron Parameters in Newborns

**DOI:** 10.3390/ijerph16111996

**Published:** 2019-06-05

**Authors:** Magdalena Chełchowska, Tomasz M. Maciejewski, Joanna Mazur, Joanna Gajewska, Anastasiya Zasimovich, Mariusz Ołtarzewski, Jadwiga Ambroszkiewicz

**Affiliations:** 1 Department of Screening and Metabolic Diagnostics, Institute of Mother and Child, Kasprzaka 17a, 01-211 Warsaw, Poland; joanna.gajewska@imid.med.pl (J.G.); mariusz.oltarzewski@imid.med.pl (M.O.); jadwiga.ambroszkiewicz@imid.med.pl (J.A.); 2 Department of Obstetrics and Gynecology, Institute of Mother and Child, Kasprzaka 17a, 01-211 Warsaw, Poland; dyrektor.naczelny@imid.med.pl (T.M.M.); tooshee@gmail.com (A.Z.); 3 Department of Child and Adolescent Health, Institute of Mother and Child, Kasprzaka 17a, 01-211 Warsaw, Poland; joanna.mazur@imid.med.pl

**Keywords:** hepcidin, erythropoietin, iron, soluble transferrin receptor, cord blood, tobacco smoke

## Abstract

The aim of this study was to assess the influence of active tobacco smoke exposure in utero on the concentration of hepcidin and selected iron markers in umbilical cord blood and to evaluate the relationships between these parameters. Newborns of smoking mothers had significantly lower concentrations of serum hepcidin (*p* < 0.001), iron, and ferritin (*p* = 0.043; *p* = 0.042, respectively), but higher levels of erythropoietin (EPO, *p* < 0.001) and soluble transferrin receptor (sTfR, *p* = 0.011) compared with newborns of non-smoking women. Negative correlations between cotinine and the number of cigarettes smoked per day with hepcidin serum level (r = −0.33, *p* = 0.033, r = −0.32, *p* = 0.041, respectively) and EPO (r = 0.47, *p* = 0.002; r = 0.46, *p* = 0.003, respectively) were found. Univariate analysis defined for the whole group of children revealed significant associations between the concentration of hepcidin and other iron status parameters. In the models estimated separately for smokers and non-smokers, we found relations between the level of hepcidin and erythropoietin (B = −0.23, *p* = 0.004; B = −0.46, *p* = 0.01, respectively). In the multivariate regression model, a negative association between hepcidin and EPO concentrations in the whole group of newborns (β = −0.53; *p* = 0.001) and in the group of smokers (β = −0.57; *p* = 0.011) was confirmed. The present study shows significant relations between smoking during pregnancy and hepcidin levels in children born at term. Decreased cord serum concentrations of hepcidin associated with high erythropoietin levels suggest induced fetal erythropoiesis, probably due to the hypoxic effects imposed by maternal smoking.

## 1. Introduction

Anemia during pregnancy is a public health problem that occurs not only in developing countries but also developed countries, including Poland [1,2,3]. It is a danger for pregnant women as well as a risk factor for child development disorders both in the pre- and postnatal period. Studies conducted in recent years indicate a relationship between anemia and preterm birth, fetal hypotrophy, and low birth weight [4,5]. Iron endowment at birth is particularly important because during the first months of life (up to 6 months) the child draws from iron stores accumulated in the third trimester of pregnancy [6]. Some authors observed correlations between iron levels and iron availability in newborns and nervous system development and cognitive skill development during adolescence [7].

Hepcidin antimicrobial peptide (HAMP) plays a key role in iron metabolism. HAMP is a protein hormone with antibacterial and antifungal activity inhibiting intestinal iron absorption and iron release from macrophages of the reticuloendothelial system [6,8]. The literature highlights the role of maternal as well as fetal hepcidin in the regulation of maternal-fetal iron transport through placental syncytiotrophoblast cells [4,9,10]. Fisher and Nemeth [6] suggest that maternal hepcidin can regulate the amount of iron that is presented to the placenta for uptake, while fetal hepcidin regulates iron export from the placenta into fetal circulation. This hormone combines with ferroportin, the only known iron protein exporter found on the surface of placental cells, enterocytes, macrophages, and hepatocytes, causing its internalization and degradation, which results in reduced iron release from cells into the blood [6]. It was found that hepcidin mRNA expression decreases in hypoxia states. Therefore, its inhibitory effect on iron absorption and recirculation is abolished, and as a result its availability grows for increased erythropoiesis in hypoxic state cells [8,11]. Hypoxia is a signal inducing the production of erythropoietin (EPO) glycoprotein, whose main function is the stimulation of erythropoiesis processes in the bone marrow. In the case of chronic tissue hypoxia, a compensatory mechanism is activated increasing the concentration of EPO in the blood, which increases the production of erythrocytes, active oxygen carriers, which improves tissue oxygenation [12].

Animal studies showed that the administration of erythropoietin via injection significantly decreased hepcidin gene expression in the liver [13]. On the other hand, in vitro studies showed that at low erythropoietin concentrations, hepcidin secreted in larger amounts inhibited the proliferation and survival of erythrocyte precursor cells, leading to anemia [14].

Iron transport in the circulation is carried out by transferrin, which donates iron to cells through its interaction with a specific membrane receptor—transferrin receptor (TfR). The transferrin receptor is present in the membranes of almost all cells of the body. In addition to mature erythrocytes, it is found in the largest amounts in the cell membranes of the placenta, liver, and bone marrow. Transferrin receptors can be measured in the serum as a soluble truncated monomer (sTfR) of tissue receptor and their concentration is related to the total amount of cell membrane bound transferrin receptor. sTfR is one iron status indicator and its level increases in serum not only in cases of iron deficiency but also in cases of intense erythropoiesis [15,16,17].

In pregnant women who smoke, the reduced oxygen transfer resulting from tobacco use, along with the physiologically increased use of oxygen in pregnancy, may result in impaired iron homeostasis. [3,18]. Depletion of maternal iron stores due to induced erythropoiesis may also induce hypoxia in the fetus, and consequently, lead to a disturbance of iron metabolism in the newborn [15,19,20]. Although in Poland 15–20% of women actively smoke tobacco during pregnancy, there has been no systematic research confirming the association between hypoxia resulting from mother’s smoking and iron metabolism in a child [21,22].

The aim of this study was to assess the relation of active tobacco smoke exposure in utero with the concentration of hepcidin and selected iron markers in umbilical cord blood. We studied the possible associations between iron parameters in newborns and markers estimating the intensity of cigarette smoking of mothers. We also investigated correlations between hepcidin and other indicators of iron status in newborns.

## 2. Materials and Methods

The study was in line with the ethical principles of the Declaration of Helsinki for Human Research and was approved by the Ethics Committee of the Institute of Mother and Child in Warsaw, Poland (Decision No. 9/2010). All mothers who agreed to participate in the study signed informed consent forms for the analysis of cord blood samples.

### 2.1. Participants

The neonates enrolled in this study were born to 80 women, who were patients of the Institute of Mother and Child in Warsaw between 2013 and 2015. The study included a consecutive series of 40 newborns of active smokers who smoked a minimum of 5 cigarettes per day throughout their pregnancy and smoked a minimum of 2 years before conception, and a series of 40 newborns of non-smokers of similar age and age of gestation, who had never smoked and were not exposed to environmental tobacco smoke during their pregnancy (smoking spouse or co-workers). History of smoking was obtained by direct questioning of the pregnant women and classification was confirmed by measuring cotinine (the major metabolite of nicotine) concentration in umbilical cord blood. The cut-off value of 13.7 μg/L was considered the limit between the non-smokers and the smokers, in accordance with Jarvis et al. [23]. All neonates were born to healthy women who did not have medical problems that are known to affect iron homeostasis at the time of enrollment. Multiple pregnancy, birth defects detected during pregnancy, assisted reproduction, delivery complications, and prolonged labor were exclusion factors from the study. The mothers remained on a mixed diet, and reported taking standard vitamins with iron and folate during pregnancy (average 400–800 mcg of folate, 15–60 mg of ferrous fumarate daily). A detailed description of the study population and data of the mothers’ iron status was reported in our previous publication [18].

Pre-pregnancy body mass index (BMI) was calculated as pre-pregnancy self-reported body weight (kg) divided by height squared (m^2^). Newborn infants were evaluated in the first 24 h of life. Neonatal length, head circumference, and weight were determined using a measuring board to the nearest 0.1 cm and a calibrated scale to the nearest 10 g.

### 2.2. Blood Sampling and Biochemical Analysis

As reported previously, mixed venous and arterial umbilical cord blood samples (10 mL) were collected at the time of delivery and before the separation of the placenta. Blood in EDTA-containing tubes was analyzed immediately for the determination of hemoglobin (Hb) using a Pentra 60 automated hematology analyzer (HORRIBA ABX, Montpellier, France). In order to obtain serum, the blood was centrifuged at 2500× g at 4 °C for 10 min, and was stored in small portions for subsequent biochemical analysis. The serum iron (Fe) level was measured by colorimetric test with FerroZine/acorbate reagennts, and ferritin (Ft) was determined by the electrochemiluminescence (ECLIA) method using commercially available kits on Cobas Integra analyzer (ROCHE, Basel, Switzerland). Serum hepcidin (bioactive hepcidin-25 molecule), erythropoietin, and soluble transferrin receptor values were determined by immunoassay (DRG Diagnostics, Marburg, Germany). The limit of detection was 0.153 ng/mL for hepcidin, 1.1 mIU/mL for EPO, and 0.02 mg/L for sTfR. The intra- and inter-assay coefficients of variation (CVs) were less than 5.7% and 9.5% for hepcidin, 8.4% and 8.8% for EPO, and 6.0% and 7.0% for sTfR, respectively. Cotinine levels were evaluated by immunoenzymatic method using a commercially available kit (Calbiotech Inc., Spring Valley, CA, USA) with a detection limit of 1.0 ng/mL.

### 2.3. Statistical Analysis

Data were processed using SPSS statistical software version 17.1 (SPSS INC., Chicago, IL, USA). Prior to statistical analysis, the Kolmogorov-Smirnov method was used to test distribution normality. The results were presented as means with standard deviation (SD) for normally distributed data or median with interquartile range (25th–75th percentiles) for non-normally distributed variables. In the smoking and non-smoking groups, the baseline characteristics were compared using the Student *t*-test or Mann-Whitney *U* test depending on the assumptions. The Chi-squared test was used for comparing categorical variables. For simple correlation analysis, Pearson’s or Spearman’s (depending on distribution) correlation coefficients were calculated to evaluate the relationships between markers for determining the intensity of mothers’ smoking and the studied newborns’ iron parameters. Univariate and multivariate regression models with hepcidin concentration as the dependent variable were evaluated to examine the potential impact of other iron parameters. The results were presented as the value of β standardized regression coefficient, B unstandarized regression coefficient with 95% confidence interval, and change in R-squared coefficient after each variable was entered. Models were estimated separately for smokers and non-smokers as well as for the whole group. The significance level was set at a *p* value of <0.05.

## 3. Results

The neonatal general characteristics and obstetric data of 80 subjects enrolled in this study are shown in Table 1. There were no significant differences in the maternal characteristics between group of the smokers and the non-smokers with respect to age, race, body mass index, mode of delivery, and declared supplement intake. In the group of smokers, the average number of cigarettes smoked per day was 8.8 and the mean duration of the habit before conception was 8.6 years. Among the neonatal parameters, mean birth weight (*p* < 0.001) body length (*p* = 0.009) respectively), and head circumference (*p* < 0.001) of the neonates born to smoking mothers were significantly lower than the newborns of non-smokers. No differences were observed in the Apgar score, fetal sex, and gestational age in these groups.

A summary of the studied newborns iron status is presented in Table 2. The newborns of smoking mothers had significantly lower concentrations of serum hepcidin, iron and ferritin, but higher levels of EPO and sTfR compared with newborns of non-smoking women. There were no significant differences in hemoglobin concentrations between the studied groups.

Table 3 shows the results of correlation studies between the tested iron parameters in cord blood and markers for determining the intensity of mother’s smoking. The hepcidin level in the infants of smokers was negatively related with maternal serum cotinine and the number of cigarettes smoked per day, while positive correlations were observed in the case of EPO concentrations and markers determining the intensity of maternal smoking. The number of cigarettes smoked per day was positively correlated with infant sTfR concentrations. We also found a statistically significant negative correlation between cord blood serum iron concentration and the number of years of smoking before conception. Other possible correlations were not statistically significant.

**Figure 1:**
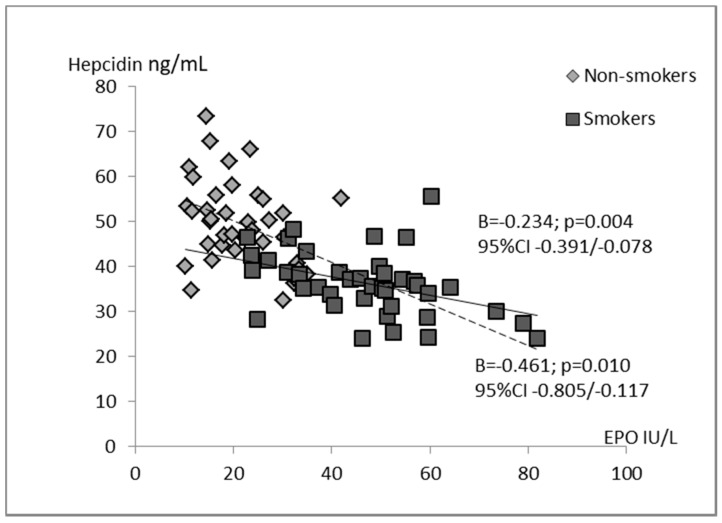
Univariate regression analyses of hepcidin with erythropoietin in the groups of smoking women’s newborns (*n* = 40) and non-smoking women’s newborns (*n* = 40).

**Figure 2:**
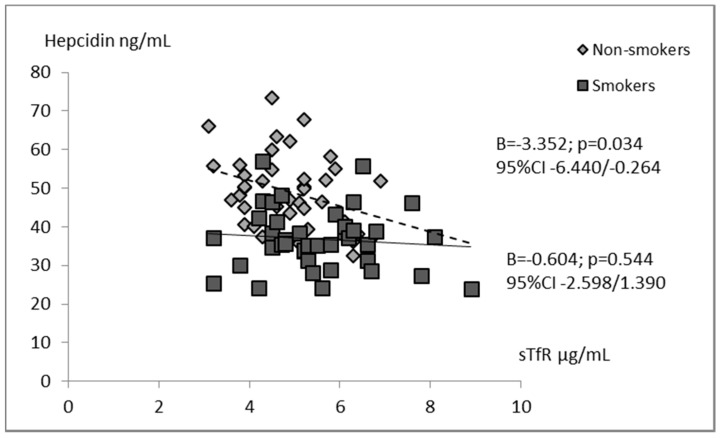
Univariate regression analyses of hepcidin with soluble transferrin receptor in the groups of smoking women’s newborns (*n* = 40) and non-smoking women’s newborns (*n* = 40).

Univariate analysis defined for the whole group of children indicated that hepcidin concentration was significantly associated with all studied iron status parameters. Negative relations between the levels of hepcidin and EPO (B = −0.401; *p* = 0.000), sTfR (B = −2.955; *p* = 0.003), and hemoglobin (B = −2.407; *p* = 0.036) were found. In contrast, hepcidin correlated positively with iron (B = 0.693; *p* = 0.033) as well as ferritin (B = 0.091; *p* = 0.035). In the models estimated separately for smokers and non-smokers, we revealed associations between concentrations of hepcidin and erythropoietin (Figure 1). Additionally, in the tobacco abstinent group, hepcidin was inversely correlated with sTfR level (Figure 2). However, in separate models for newborns of smoking and non-smoking mothers, no significant relationships between infants’ hepcidin levels and other iron status markers were observed.

Table 4 contains the results of multivariate regression analysis, confirming a negative association between hepcidin and erythropoietin concentrations in the whole group of newborns (β = −0.531; *p* = 0.001) and the group of smokers (β = −0.565; *p* = 0.011). In these subjects, EPO explained 44.6% and 31.9% of the hepcidin variance, respectively. We did not find this relation in the infants of tobacco abstinent women. A tendency towards a positive association between cord blood hepcidin concentrations and the pregnant women’s smoking habit was also observed (β = 0.178; *p* = 0.073).

## 4. Discussion

The presented lower hepcidin concentrations in the newborn’s cord blood of smoking mothers coexisting with higher levels of erythropoietin and sTfR could be the result of hypoxia in the fetus induced by smoking by the mother. Chronic exposure to the carbon monoxide contained in tobacco smoke causes carboxyhemoglobinemia, thus limiting hemoglobin availability to the fetus. Additionally, by increasing catecholamine levels in the mother’s blood, nicotine causes increased vascular resistance and excessive vasospasms, which limit blood flow through the placenta and causes insufficient gas exchange in the fetal-placental unit [24]. Hypoxia is intensified by cyanide compounds contained in tobacco smoke that impair the oxidative mechanism of the fetus. Finally, a recent report showed a positive relation between maternal smoking and an increase in lead concentration in the fetal unit [25]. Hypoxia associated with chronic lead exposure is a result of both interfering with heme biosynthesis and by decreasing red blood cell survival [26]. Pb can also cause potentially hypoxic effects due to its pro-inflammatory and pro-oxidative properties [27]. Oxygen transfer disorders, resulting from smoking together with increased oxygen consumption during pregnancy, can lead to increased fetal erythropoiesis, reduced iron transport through the placenta, increased iron consumption, and depletion of iron stores [3,28]. Our previous studies conducted in a group of women in the third trimester of pregnancy showed that smoking significantly affects iron status in mothers and may cause hypoxia in the fetus [18].

The literature data on iron status in the cord blood of newborns of smoking mothers are not conclusive. The study of Pateva et al. [28] demonstrated that children of smoking mothers had significantly lower total iron levels compared with newborns of non-smokers, while Secovanic et al. [25] observed increased levels of iron in the cord blood of smokers. In the current study, we observed significantly lower iron levels in children of smoking women; however, it was within the normal range for umbilical cord blood [29,30]. This may be due to the increased iron use for increased erythropoiesis in the blood of children of smoking mothers. Maternal iron status may also affect the amount of iron delivered to the fetus. In women who smoked during pregnancy, lower levels of hemoglobin, hematocrit and iron were observed with longer smoke exposure and more cigarettes smoked per day [31,32,33]. Some authors found significantly higher Hb levels in the newborns of smokers than in the non-smokers’ children, while others did not observe any differences between the groups [3,18,33]. We also did not confirm a significant association of smoking during pregnancy with hemoglobin concentration in umbilical cord blood. In the studied group, Hb concentrations were slightly higher, but this difference was not statistically significant. Under conditions of iron deficiency observed in smoking mothers, the expression of receptors for this element in the placenta increased and as a result there was no decrease in fetal hemoglobin concentrations [2,4].

Since the main task is to maintain normal hemoglobin levels for the fetus, in a situation of decreased iron metabolism parameters in the mother, erythropoiesis is stimulated in the fetus, which is associated with increased erythropoietin levels in fetal blood [11,12,24]. The literature data showed that erythropoietin concentrations in the umbilical blood of children of smoking mothers was higher than in the offspring of non-smokers [24,34]. These observations were confirmed by our results. In our study, similarly to the study of Sazak et al. [24], erythropoietin levels were closely related to cotinine concentrations in the cord blood and the number of cigarettes smoked by the mother.

As a result of increased erythropoiesis and depleted iron serum levels in the fetus, iron stores are mobilized for the production of erythrocytes, which leads to decreased protein storage of ferritin. Probably, due to the increase in the number of erythroid progenitor cells, that express high number of transferrin receptors, the concentrations of sTfR in the blood increases [16].

In the current study, ferritin concentrations in umbilical cord blood of children of smoking mothers were significantly lower and sTfR levels were significantly higher than in the children of non-smokers, which is consistent with other authors observations [16,28]. High concentrations of erythropoietin and sTfR and the accompanying decrease in total iron in the blood of newborns of smoking mothers may indicate hypoxia in the fetus.

The effect of chronic hypoxia caused by smoking on hepcidin levels in umbilical cord blood had not been systematically investigated so far. Briana et al. [11] did not observe differences between hepcidin concentrations in the cord blood of newborns of smoking and non-smoking mothers. The hepcidin values we obtained were within the ranges observed by other authors, but in the group of children of smokers, these values were significantly lower than in the group of non-smokers [7,20,35,36,37]. These differences may result from the fact that our research was conducted in a homogeneous group of healthy children, born at term with normal birth weight, while the study of Briana et al. [11] included a group of newborns with features of intarauterine growth restriction (IUGR). In newborns with IUGR, the mechanism of hepcidin secretion is disturbed on the one hand by the inhibitory effects of hypoxia and iron deficiency and on the other hand by the induction of chronic inflammation.

The negative correlation demonstrated in our study between hepcidin concentrations and cotinine levels in cord blood, and the number of cigarettes smoked by the mother per day suggest that the level of this hormone can be associated with the exposure dose to tobacco smoke. In addition, the negative correlation of hepcidin with erythropoietin levels may indicate developing hypoxia in the fetus. Similar correlations were observed in the cord blood of low birth weight infants and in newborns not exposed to tobacco smoke but with documented anemia [7,20,38].

A limitation of the study included the fact that the research scheme used is not typical for a case-control study with retrospective assumptions [39]. This is a prospective comparison of cohorts exposed and not exposed to the effect of one factor, in this case tobacco smoking. The recruitment method used does not indicate the percentage of smokers representing the normal population of pregnant women. Nevertheless, groups of smokers and non-smokers that did not differ in size and other basic characteristics could be compared.

The study group should be larger to grant sufficient power to detect strong associations with statistical significance; however, both groups were ethnically homogenous, comparable in fetal sex, gestational age, and mode of delivery [10,37]. Another limitation is that we did not measure any inflammatory parameters (e.g., IL6—important inducer of hepcidin) in cord blood, but we included healthy neonates without symptoms of inflammation. In addition, some authors did not confirm the correlation between hepcidin concentrations and the markers of inflammation measured in umbilical cord blood [37,40,41]. We were unable to measure the ferroportin level which is considered to be the main iron transporter from syncytiotrophoblast into the fetal circulation [6,42]. Finally, we did not estimate maternal iron dietary intake, but our population consisted of healthy, non-anemic women who did not use elimination diets and declared receiving multivitamin supplements as part of routine prenatal care. An advantage that compensates for the above limitations is the ability to assess iron markers in newborns of mothers actively smoking more than 5 cigarettes per day throughout the entire pregnancy.

## 5. Conclusions

The present study shows significant relations between smoking during pregnancy and hepcidin levels in children born at term. Decreased cord serum concentrations of hepcidin associated with high erythropoietin levels suggest induced fetal erythropoiesis, probably due to the hypoxic effects imposed by maternal smoking.

## Figures and Tables

**Table 1:** General characteristics of the study subjects (*n* = 80).

Group	Smokers (*n* = 40)	Non-Smokers (*n* = 40)	*p*-Value
Newborn Characteristics			
Gestational age, weeks	39.0 ± 1.0	39.3 ± 0.9	0.384
Apgar 1th min score	10 (9–10)	10 (10–10)	0.511
Apgar 5th min score	10 (10–10)	10 (10–10)	0.576
Male/Female, %	46/54	38/62	0.420
Birth weight, g	3124 ± 427	3517 ± 446	<0.001
Birth body length, cm	54.5 ± 2.7	55.9 ± 2.5	0.009
Head circumference, cm	34.0 ± 1.7	35.0 ± 1.1	<0.001
Cotinine, µg/L	84.5 (56.3–112.1)	0	-
Maternal Characteristics			
Age, years	28.8 ± 4.5	30.3 ± 4.8	0.120
Race: Caucasian, %	100	100	-
Mode of delivery: vaginal, %	100	100	-
Pre-gravid BMI, kg/m^2^	23.4 ± 1.3	24.1 ± 1.4	0.073
Hemoglobin, g/dL	12.2 ± 1.1	12.7 ±1.1	0.049
Smoking habit smokers/non-smokers, %	50	50	-
Number of cigarettes, day	10 (5–10)	-	-
Time of smoking before conception, year	8 (5–12)	-	-

Results are presented as means ± SD, median (25–75% interquartile range), percent (%), BMI—body mass index.

**Table 2:** Concentrations of hepcidin and selected iron parameters in newborn.

Cord Iron Status Parameters	Newborns of Smoking Mothers (*n* = 40)	Newborns of Non-Smoking Mothers (*n* = 40)	*p*-Value
Hepcidin, ng/mL	36.8 ± 7.8	49.1 ± 9.6	<0.001
Erythropoietin, mIU/mL	46.6 ± 14.6	22.2 ± 8.4	<0.001
Ferritin, ng/mL	137.7 ± 26.2	150.1 ± 27.7	0.042
Iron, µmol/L	15.5 ± 4.0	17.3 ± 3.8	0.043
Haemoglobin, g/dL	16.3 (16.2–16.9)	16.0 (15.8–16.4)	0.063
Soluble transferrin receptor, μg/mL	5.5 ± 1.3	4.9 ± 1.0	0.011

Results are presented as means ± SD, median (25–75% interquartile range).

**Table 3:** Simple correlations between iron status parameters in the cord blood of newborns and markers for determining the intensity of mother’s smoking (*n* = 40).

Cord Iron Status Parameters	Cotinine Level		Number of Cigarettes/Day		Time of Smoking Before Conception	
	r	*p*-Value	r	*p*-Value	r	*p*-Value
Hepcidin ^a^	−0.334	0.033	−0.320	0.041	−0.128	0.425
Erythropoietin ^a^	0.474	0.002	0.456	0.003	0.045	0.778
Ferritin ^a^	−0.250	0.115	0.064	0.692	−0.087	0.589
Iron ^a^	−0.262	0.099	−0.055	0.731	−0.332	0.034
Hemoglobin ^b^	0.185	0.247	0.226	0.155	−0.018	0.912
Soluble transferrin receptor ^a^	0.192	0.228	0.646	0.000	0.023	0.883

^a^ Pearson’s correlation coefficient; ^b^ Spearman’s correlation coefficient.

**Table 4:** Multivariate regression of hepcidin with iron status parameters and markers for determining the intensity of mother’s smoking in the whole group of studied newborns, and in the subgroups of smoking women’s newborns and non-smoking women’s newborns.

Parameter	All Newborns (*n* = 80)		Newborns of Smoking Mothers (*n* = 40)		Newborns of Non-Smoking Mothers (*n* = 40)	
	β	*p*-Value	β	*p*-Value	β	*p*-Value
Erythropoietin	−0.531	0.001	−0.565	0.011	−0.316	0.101
Ferritin	0.123	0.212	0.205	0.231	0.085	0.629
Iron	−0.049	0.645	−0.098	0.601	0.017	0.925
Hemoglobin	0.115	0.288	0.323	0.103	−0.028	0.873
Soluble transferrin receptor	−0.100	0.277	0.129	0.526	−0.227	0.165
Smoking status (no = 0; yes = 1)	0.178	0.073	-	-	-	-
Cotinine	-	-	0.007	0.972	-	-
Number of cigarettes/day	-	-	−0.242	0.350	-	-
R^2^%	46.6		31.9		22.5	
